# Enhanced Cartilaginous Tissue Formation with a Cell Aggregate-Fibrin-Polymer Scaffold Complex

**DOI:** 10.3390/polym9080348

**Published:** 2017-08-08

**Authors:** Soojin Lee, Kangwon Lee, Soo Hyun Kim, Youngmee Jung

**Affiliations:** 1 Biomaterials Research Center, Korea Institute of Science and Technology, 5, Hwarang-ro 14-Gil, Seoungbuk-gu, Seoul 02792, Korea; dltnwls830@kist.re.kr (S.L.); soohkim@kist.re.kr (S.H.K.); 2 Program in Nanoscience and Technology, Graduate School of Convergence Science and Technology, Seoul National University, Seoul 08826, Korea; kangwonlee@snu.ac.kr; 3 Advanced Institutes of Convergence Technology, Gyeonggi-do 16229, Korea; 4 KU-KIST Graduate School of Converging Science and Technology, Korea University, Seoul 02841, Korea; 5 Division of Bio-Medical Science & Technology, KIST School, Korea University of Science and Technology, Seoul 02792, Korea

**Keywords:** cell aggregate, hydrogel, poly (lactide-*co*-caprolactone), cartilage regeneration, hanging drop method

## Abstract

Cell density is one of the factors required in the preparation of engineered cartilage from mesenchymal stem cells (MSCs). Additionally, it is well known for having a significant role in chemical and physical stimulations when stem cells undergo chondrogenic differentiation. Here, we developed an engineered cartilage with a cell aggregate-hydrogel-polymer scaffold complex capable of inducing the effective regeneration of cartilage tissue similar to natural cartilage while retaining a high mechanical strength, flexibility, and morphology. Cell aggregates were generated by the hanging drop method with rabbit bone marrow stromal cells (BMSCs), and poly (lactide-*co*-caprolactone) (PLCL) scaffolds were fabricated with 78.3 ± 5.3% porosity and a 300–500 μm pore size with a gel-pressing method. We prepared the cell aggregate-fibrin-poly (lactide-*co*-caprolactone) (PLCL) scaffold complex, in which the cell aggregates were evenly dispersed in the fibrin, and they were immobilized onto the surface of the polymer scaffold while filling up the pores. To examine the chondrogenic differentiation of seeded BMSCs and the formation of chondral extracellular matrix onto the complexes, they were cultured in vitro or subcutaneously implanted into nude mice for up to eight weeks. The results of the in vitro and in vivo studies revealed that the accumulation of the chondral extracellular matrices was increased on the cell aggregate-fibrin-PLCL scaffold complexes (CAPs) compared to the single cell-fibrin-PLCL scaffold complexes (SCPs). Additionally, we examined whether the mature and well-developed cartilaginous tissues and lacunae structures typical of mature cartilage were evenly distributed in the CAPs. Consequently, the cell aggregates in the hybrid scaffolds of fibrin gels and elastic PLCL scaffolds can induce themselves to differentiate into chondrocytes, maintain their phenotypes, enhance glycosaminoglycan (GAG) production, and improve the quality of cartilaginous tissue formed in vitro and in vivo.

## 1. Introduction

The principal function of articular cartilage, the dense connective tissue of the diarthrodial joints, is to provide a lubricated surface as well as to bear high stress and friction loads in the body [1,2]. Articular cartilage composed of the extracellular matrix (ECM) with chondrocytes has a less intrinsic capacity for self-healing, because it does not have blood vessels, nerves, or lymphatics for promoting wound healing. Thus, when articular cartilage is damaged, it cannot repair itself spontaneously or rapidly unlike other tissues in the human body. This gives rise to severe consequences such as pain or reduced mobility caused by further degeneration [3,4,5,6].

Many studies have been performed in an effort to overcome the above-mentioned limitations of previously known therapies for cartilage regeneration. Thus, a treatment method for deep cartilage defects in the knee with autologous chondrocyte transplantation has been reported [7,8,9]. Because the above method has been proved successful in obtaining regenerative cartilage tissue, which is relatively close to natural cartilage, by culturing autologous chondrocytes, clinical trials using autologous chondrocyte transplantation (ACT) have been steadily rising in the United States and Northern Europe. However, because ACT injects cultured chondrocytes in a suspension directly into a cartilage defect area, ACT has several limitations, including a difficulty in obtaining a sufficient amount of chondrocytes [10], delamination, and underfilling of the defect. The injected cells are easily washed out after transplantation, and as a result, it is difficult to maintain a high cell density in the defect area [11].

Although many studies have been conducted on cartilage regeneration using techniques that include tissue engineering and ACT, some limitations still remain, such as the formation of fibrocartilage generated from the transplanted chondrocytes, and a reduced mechanical strength of the newly formed tissues compared to native cartilage, which is problematic in terms of the mid-/long-term durability of the cartilage regeneration [12,13,14,15,16,17]. The regeneration process must induce a considerable glycosaminoglycan (GAG) production to generate functional engineered cartilage tissue, in other words, to construct a structure which can bear loads in the human body [18] and avoid fibrocartilage formation [19].

To mimic better physiological tissues, three-dimensional (3D) cell cultures have been studied because cellular functions and responses in tissues are often lost in two-dimensional cell cultures [20]. In many studies, they have used the pellet culture or micro mass culture technique to induce the chondrogenic differentiation of cells or to form cartilage-like tissues. By using these culture systems, they supply a three-dimensional morphology of cartilage to cells, which has an important role in promoting cell–matrix interactions and cell–cell interactions during chondrogenesis. Furthermore, the phenotypic features of cartilage are closely related to its three-dimensional matrix of collagen and proteoglycans, which are lost in monolayer cultures [21,22].

High-density cell cultures, such as the above-mentioned methods, are widely used to induce cells to differentiate and to maintain the chondrocyte phenotype by providing the proper environment to the cells [23]. In this study, we developed cell aggregates with bone marrow stromal cells (BMSCs) that have a high density using a hanging drop culture. The cell aggregates spontaneously formed a three-dimensional structure during the culture in vitro. After that, we seeded the cell aggregates onto hybrid scaffolds of fibrin gels and elastic poly (lactide-*co*-caprolacton) (PLCL) scaffolds, which we have previously fabricated [24]. These scaffolds, called cell aggregate-fibrin-PLCL scaffold complexes (CAPs), were then used to evaluate the feasibility of chondrogenic differentiation. We also compared the CAPs to conventional single cell-fibrin-PLCL scaffold complexes (SCPs) to evaluate their capacity for cartilage regeneration in vitro and in vivo.

## 2. Materials and Methods

### 2.1. Preparation of the PLCL Scaffolds

PLCL was synthesized and subjected to a gel-pressing method to fabricate sheet-form scaffolds, as described elsewhere [25]. Briefly, PLCL (50:50) was synthesized at 150 °C for 24 h in the presence of Stannous Octoate (1 mmol, Sigma, St. Louis, MO, USA) as a catalyst. Dissolved PLCL (5% *w/v*, in Chloroform) was mixed homogeneously with approximately 300 μm sized salts to form the PLCL gels. The residual solvents were removed by placing the samples under vacuum for an additional 24 h. The salts were then leached out by immersing the samples in distilled, deionized water and subjecting them to constant shaking for 3 days. The resulting scaffolds (disk type, diameter = 9 mm and thickness = 3 mm) were freeze-dried for 24 h, and then sterilized with ethylene oxide gas. The porosity of the scaffolds was measured by a mercury intrusion porosimeter (Auropore IV; MicroMeritics, Norcross, GA, USA).

### 2.2. Bone Marrow Stromal Cell Isolation and Culture

Cell isolation was conducted using a modification of the method described by Dong et al. and Cho et al. [25,26]. Briefly, bone marrow cells (BMCs) obtained from the femora and tibiae of New Zealand White rabbits (250–400 g) were treated with Ficoll–Paque density gradient reagent (Sigma; St. Louis, MO, USA) for separating the bone marrow mononuclear cells (BMMNCs), and the isolated cells were cultured with culture medium (Dulbecco’s Modified Eagle Medium/Nutrient Mixture Ham-12 (DMEM/F12, Sigma) containing 10% Fetal Bovine Serum (FBS, US biotechnologies, Logan, UT, USA) and 1% penicillin-streptomycin (P-S, Gibco Life Technologies, Carlsbad, CA, USA)). The medium was replaced every two or three days to remove any non-adherent cells. Prior to preparing the cell aggregates, the BMSCs were labeled with the Vybrant^®^ CFDA SE Cell Tracer Kit (Molecular Probes, Eugene, OR, USA) at a concentration of 1 µM CFDA/mL phosphate-buffered saline (PBS) (Sigma).

### 2.3. Preparation of the Cell Aggregates with the Hanging Drop Method

The cultured BMSCs (passage number equal to 2) were collected by trypsin (Gibco BRL, Carlsbad, CA, USA) treatment and then resuspended in chondrogenic medium (DMEM, 1 mM Sodium Pyruvate (Sigma), 100 nM Dexamethasone (Sigma), 20 µg/mL Proline (Sigma), 37.5 µg/mL ascorbic acid 2-phosphate (Sigma), 1% P-S, 1% FBS, 1× insulin-transferrin-selenium (ITS+) (BD Bioscience, Allschwil, Switzerland), and 10 ng/mL TGF-β_1_ (R&D Systems, Minneapolis, MN, USA); 3–5 × 10^5^ cells/mL). Drops of 20 µL containing 6000–10,000 BMSCs suspended in culture medium were placed on the inner side of the lid of 100 mm tissue culture dishes (Corning, Corning, NY, USA). The dishes were then filled with PBS to avoid a loss of nutrients through evaporation, and incubated for 7 days in a hanging drop at 37 °C in a CO_2_ incubator [27].

### 2.4. In Vitro and In Vivo Studies of the Cells-Scaffold Complexes with Fibrin Gels and PLCL Scaffolds

**Figure 1:**
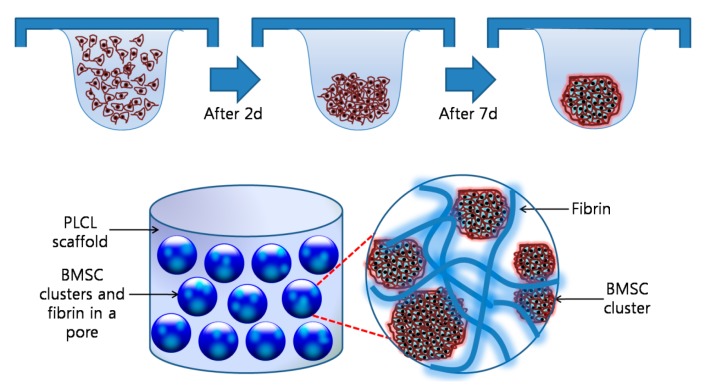
An overall schematic illustration of the preparation of a cell aggregate by the hanging drop method and the structure of a cell aggregate-fibrin-polymer scaffold complex. PLCL, poly (lactide-*co*-caprolacton); BMSC, bone marrow stromal cell.

Figure 1 shows an overall schematic illustration of the preparation of a cell aggregate by the hanging drop method and the structure of a CAP. To prepare the CAPs, a suspension of cell aggregates (about 2 × 10^6^ cells/scaffold) was dispersed in 35 µL of fibrinogen solution (90 mg of fibrinogen, 60 U of factor XIII, and 1000 KIU aprotinin/mL) provided in a fibrin glue kit (Greenplast^®^; Yongin, Korea), and then mixed homogeneously. After the addition of 35 µL of thrombin solution (20 IU/mL of 0.6% (*w/v*) calcium chloride solution), the mixture was rapidly incubated in a fibrin-PLCL scaffold (9 mm diameter; disk type). In addition, a single cell suspension in chondrogenic medium was inoculated in the same conditions as the cell aggregates for the formation of the SCPs. The complexes were then allowed to stabilize in the incubator for one hour, after which chondrogenic medium was added. The complexes were then cultured for 25 days in a humidified incubator at 37 °C under 5% CO_2_ in vitro.

For the in vivo studies, CAPs or SCPs were implanted into mice to investigate the chondrogenic differentiation of the cell aggregates and the cartilaginous tissue formation in the complexes. The complexes were prepared with the same method that was used for the in vitro studies, and then implanted into the subcutaneous dorsum of seven-week-old male athymic mice (SLC, Hamamatsu, Japan). The implants were harvested for analysis after 4 or 8 weeks.

### 2.5. Evaluation of the Cells-Scaffold Complexes

#### 2.5.1. Scanning Electron Microscopy (SEM) Micrographs

The morphologies of the cell aggregates and the complexes were examined by scanning electron microscopy (SEM; Hitachi, Tokyo, Japan) at 15 kV. The samples were fixed in 4% (*v/v*) formaldehyde for 1 day, dehydrated with a graded ethanol series, and freeze-dried. The samples were coated with gold using a sputter-coater (Eiko IB3, Tokyo, Japan).

#### 2.5.2. Water Soluble Tetrazolium Salts (WST) Assay

The numbers of cells in the cells-scaffold complexes were determined using a water soluble tetrazolium salt (WST) assay (Cell counting kit-8, Dojindo, Rockville, MD, USA), and cell proliferation and viability were assayed colorimetrically [28]. Briefly, 450 µL of culture medium and 50 µL of the kit solution were added to each sample containing the same amount of cells in 48-well plates (Corning), and incubated at 37 °C in a 5% CO_2_ humidified incubator for 4 h. The resulting solution (100 µL per well) was transferred to 96-well µL plates (Corning), and analyzed using a microplate reader (VERSA max, Molecular Devices; San Diego, CA, USA) at 450 nm.

#### 2.5.3. Immunofluorescent and Histological Analysis

To appraise the cellular organization and differentiation of the cell-aggregates in the fibrin-PLCL scaffolds three-dimensionally, immunofluorescence staining was performed with the cells-scaffold complexes. The complexes cultured in vitro for 1, 7, or 21 days were washed with PBS, fixed with paraformaldehyde for 1 day, permeabilized using Target Retrieval Solution (DaKoCytomation, Santa Clara, CA, USA), washed with PBS/bovine serum albumin (BSA, Sigma), and incubated with the primary antibody mouse-anti-chicken Collagen type II (Molecular Probes; Eugene, OR, USA) in 1% PBS/BSA overnight at 4 °C. The complexes were washed and labeled with the corresponding antibody Alexa Fluor^®^-594 conjugated anti-mouse (Molecular Probes) diluted 1:1000 in 1% BSA/PBS for 1 h at room temperature. Nuclei were counterstained with 4′,6-diamidino-2-phenylindole (DAPI, Molecular Probes), which binds to the double-strained DNA by forming a stable, blue-fluorescent complex. Finally, the complexes were mounted with Gelmount (M01, Biomeda, Foster City, CA, USA) and examined with a NiKon EZ-C1 confocal laser scanning microscope (NiKon, Tokyo, Japan) [29].

For histological analysis, the cells-scaffold complexes cultured in vitro or in vivo were fixed and embedded in paraffin. The 5 µm thick sectioned species were stained with hematoxylin and eosin (H&E). The retrieved constructs were stained with Masson’s trichrome (M-T) for checking the collagen composition, and Alcian blue and Safranin O for confirming sulfated GAGs [30]. Additionally, with the sample slides, Collagen type II secreted by the seeded BMSCs was detected by immunofluorescence staining. Nuclei were counterstained with DAPI to confirm the presence of chondrocytes in the lacunae surrounded by the labeled type II collagen. The labeled tissues were examined with fluorescence microscopy (Eclipse TE2000U, Nikon, Tokyo, Japan).

#### 2.5.4. Real-Time Polymerase Chain Reaction (Real-Time PCR)

To examine the chondrogenic differentiation of the seeded BMSCs or BMSC aggregates in the complexes, real-time polymerase chain reactions were performed [31]. The complexes were homogenized in TRIzol reagent (Gibco BRL, Carlsbad, CA, USA), after which the total RNA was extracted according to the manufacturer’s instructions. Two micrograms of RNA were subsequently reversed transcribed into cDNA in a 20 μL reaction with the Omniscript^®^ System (Qiagen, Hilden, Germany). The reverse transcription reaction was done as follows: the samples were held at 37 °C for 60 min, followed by 95 °C for 5 min in a thermal cycler. The oligonucleotide primers used in the real-time PCR are listed in Table 1 [32,33]. The real-time PCR was performed with the Applied BioSystems 7500 Real Time PCR system (Applied Biosystems, Foster City, CA, USA) and the *Power* SYBR^®^ Green PCR Master Mix (Applied Biosystems, Foster City, CA, USA). The real-time PCR reactions were done with a final volume of 25 µL using 2 to 2.5 µL of cDNA as the template. The cDNA was amplified as follows: 45 cycles of 95 °C for 15 s and 55 °C for 60 s. A melting curve analysis was then conducted at the end of the cycles to make sure that the reaction produced only a single PCR product. The results were evaluated with the 7500 System SDS v1.4 software (Applied Biosystems). Glyceraldehyde-3-phosphate dehydrogenase (GAPDH) primers were used for the normalization of the samples. In the RNA extraction, RNase-free water (Qiagen Inc., Valencia, CA, USA) was included to monitor crossover contamination of the PCR, and it was used as a negative control. To ensure the quality of the data, a negative control was used in each run [32].

#### 2.5.5. Measurement of the Glycosaminoglycan (GAG) Levels

The glycosaminoglycan (GAG) levels in the complexes were determined using the dimethyl methylene blue (DMB) assay. The complexes were homogenized and digested with 1 mL of papain solution containing 125 µg/mL papain (Sigma), 100 mM phosphate buffer (Sigma), 10 mM cysteine (Sigma), and 10 mM EDTA (Sigma) at 60 °C for 18 h. The GAG concentrations were then estimated by treating the samples with DMB dye (Sigma) [34] and comparing them against a chondroitin sulfate standard curve. All of the results are presented as the means and standard deviations (SD) (*n* = 3).

### 2.6. Statistical Analysis

All quantitative results were obtained from triplicate samples and all data were expressed as the means ± standard deviations. MINITAB^TM^ (Minitab Inc., Centre County, PA, USA) was used for the statistical analysis. The samples were analyzed by one-way analysis of variance (ANOVA) followed by nonparametric LSD (Least Significant Differences) tests. A *p* < 0.05 was considered to indicate statistical significance.

## 3. Results and Discussion

### 3.1. PLCL Scaffold Characteriztion

Synthetic polymers, such as poly (ester-ether) polydioxanone (PDS), poly (ethylene glycol) (PEG), and poly (lactic-*co*-glycolic) acid (PLGA) are used as a scaffold to bear the load and to provide the three-dimensional (3D) matrix, while mesenchymal stem cells (MSCs) differentiate into chondrogenic lineages. However, PDS has poor solubility in organic solvent. PEG is a non-load-bearing scaffold, and provides no biological signals to the cells [35]. In cartilage regeneration, delivering mechanical signals to adherent cells in the body is important, and in order to transfer the improved mechanical signals more effectively, it is important that the elasticity and mechanical property of scaffold is close to those of natural cartilage [36]. Lastly, PCL is a common material to fabricate a biodegradable porous scaffold, because PCL is biocompatible while it has enough strength to bear the load [37,38].

PLCL has a flexible and elastic property which is close to natural cartilage, and it is more efficient for delivering mechanical signals to adherent cells than PCL scaffold [31]. Biodegradable porous PLCL scaffolds were fabricated by the gel-pressing method. The scaffolds had 78.3 ± 5.3%, which was measured by the mercury intrusion porosimeter, and pore size in the range of 300–500 µm. These scaffolds also had a homogeneously interconnected and open pore structure without a skin layer, and possess high elastic and flexible properties [36,39]. Moreover, the scaffolds are capable of offering a suitable environment for cell adhesion, and, in growth, maintaining mechanical integrity and delivering mechanical signals efficiently to adherent cells [40].

### 3.2. Charaterization of the Cell Aggregates

**Figure 2:**
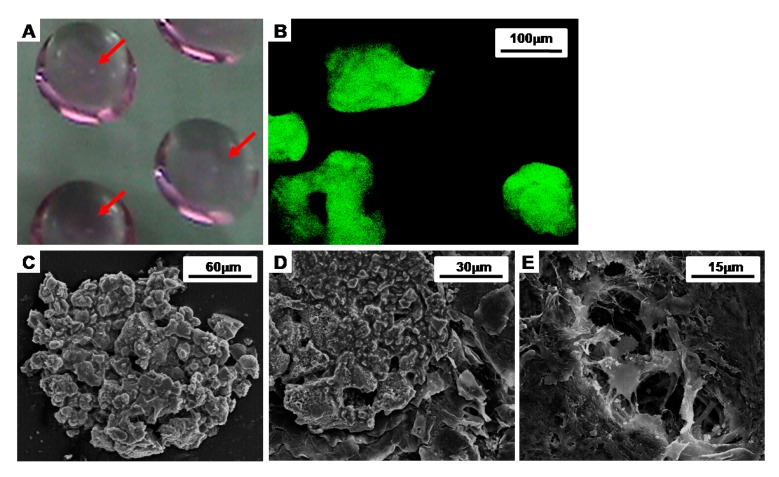
Images of the cell aggregates formed by the hanging drop method after 7 days. (**A**) An optical inverted microscope photograph of the cell aggregates; (**B**) A fluorescent microscope photograph of the cell aggregates in which the cells were labeled with CFDA cell tracer; (**C**–**E**) SEM images of a cell aggregate.

To facilitate and enhance the chondrogenic differentiation of BMSCs, we made the cell aggregates with the hanging drop method. It was observed that the BMSCs in the media drops aggregated over time (indicated by red arrow, Figure 2A). To investigate the morphology of the cell aggregates, the BMSCs were labeled with CFDA cell tracer, and the labeled cell aggregates were shown in green by a florescent microscope (Figure 2B). The cell aggregates were also analyzed by SEM, and it revealed that they formed spherical, dense cell clusters with a size of 182 ± 50 µm in diameter (Figure 2C–E).

**Figure 3:**
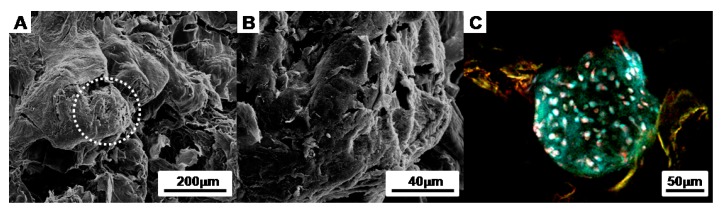
Images of a cell aggregate-fibrin-polymer scaffold complex (**A**,**B**) SEM images of a cell aggregate-fibrin-polymer scaffold complex; (**C**) a confocal microscope image of a cell aggregate-fibrin-polymer scaffold complex at 2 days after seeding (Green: CFDA labeled cells; Blue: DAPI labeled nucleic acid).

To confirm the morphology of the cell aggregates in the CAPs, we observed the surface with SEM and a florescent microscope. The images show that the cell aggregates were tightly adhered onto the fibrin-PLCL scaffolds, maintaining their morphologies (Figure 3).

### 3.3. Evaluation of Cells-Scaffold Complexes: In vitro and In vivo Experiments

**Figure 4:**
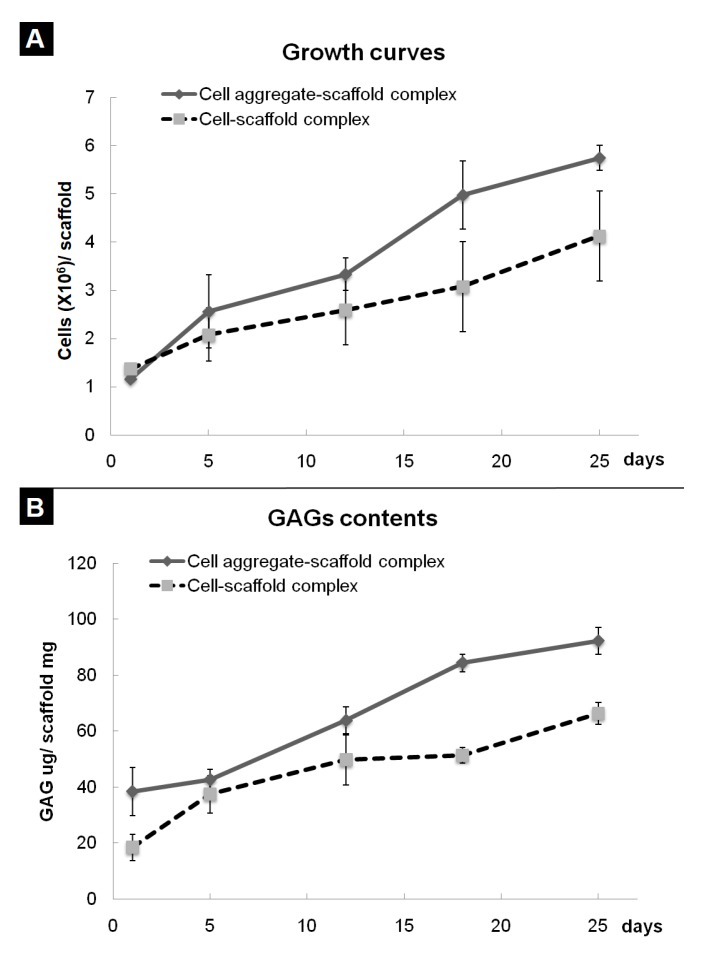
Quantitative analysis of cell growth (**A**) and glycosaminoglycan (GAG) levels (**B**) associated with cell aggregate-fibrin-polymer scaffold complexes and cell-fibrin-PLCL scaffold complexes cultured in vitro. The error bars show the standard deviations. (Cell-scaffold complex, complex seeded with single cells of BMSCs; Cell aggregate-scaffold complex, complex seeded with cell aggregates of BMSCs).

In previous research, we developed a fibrin-PLCL complex construct seeded with BMSCs [41]. Dense collagen fibrils oriented with a relatively high density of ellipsoid-shaped flattened chondrocytes, which means that a high density of BMSCs is more effective than a low density of cells [35]. To evaluate the effect of cell aggregation on the chondrogenic differentiation of bone marrow stromal cells in vitro, the cell aggregates or single cells of BMSCs were seeded onto each fibrin-PLCL scaffold and cultured for 25 days. A cell adhesion, viability, and proliferation assay was done with the WST assay. Figure 4A shows the cell growth on each fibrin-PLCL scaffold over a 25-day period. The growth data show a significant difference in the cell proliferation rate between the CAPs and SCPs. DMB assays were also performed to determine the GAG content, which is one of the markers for chondrocyte differentiation and cartilaginous tissue formation. Figure 4B shows a plot of the GAG content, and the data exhibited a similar pattern to that of the cell proliferation. Both the cell growth rate and the GAG content of the CAPs were higher than those of the SCPs, and tended to increase over time.

**Figure 5:**
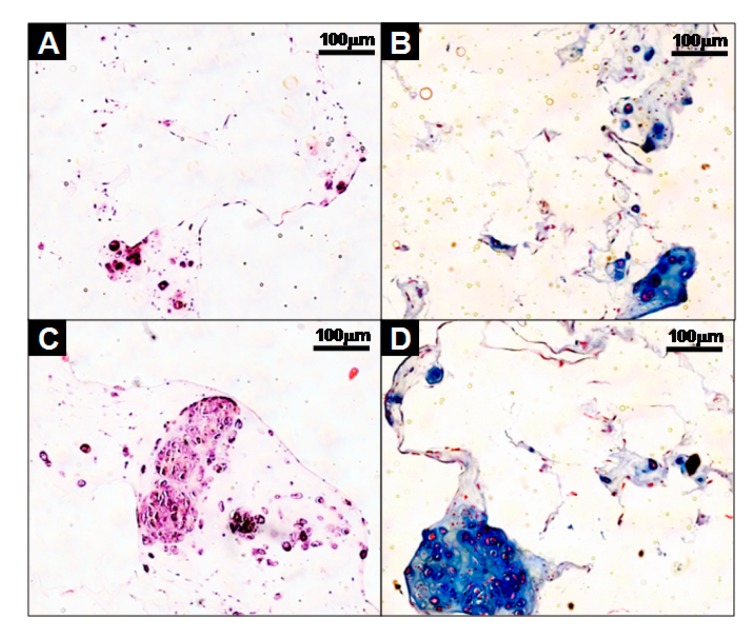
Histological studies of cells-scaffold constructs cultured for 21 days in vitro. The sections were stained with hematoxylin and eosin (H&E) (**A**,**C**), or Alcian Blue (**B**,**D**). The images show the cell-fibrin-PLCL scaffold complexes (**A**,**B**) and cell aggregate-fibrin-PLCL scaffold complexes (**C**,**D**).

Furthermore, the cells-scaffold complexes were cultured for 21 days in vitro and stained with hematoxylin and eosin (H&E) and alcian blue for histological analysis. The images of the analysis’ result are presented in Figure 5. After 21 days of culturing, there were little or no lacunae in the SCPs (Figure 5A), while the cell aggregates in the CAPs formed mature and well-developed cartilage tissue, as evident by the chondrocytes within the lacunae (Figure 5C). An amount of sulfated GAG deposition was observed in the CAPs with a strong blue color for the alcian blue staining, and mature chondrocytes within the lacunae were also observed (Figure 5D). In contrast, less sGAG content from the SCPs was stained (Figure 5B). These results show that the CAPs are more effective in forming mature and well-developed cartilaginous tissues in vitro.

**Figure 6:**
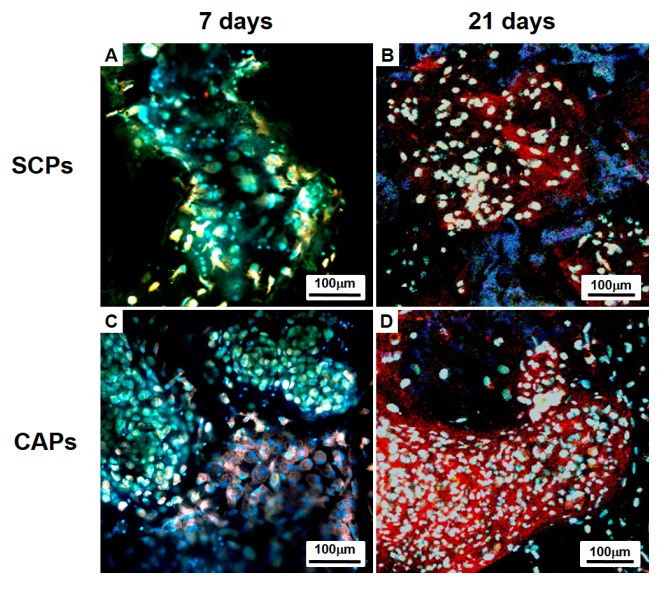
Immunofluorescence studies with a confocal microscope of the cells-scaffold constructs cultured for 7 days (**A**,**C**) or 21 days (**B**,**D**) in vitro. The complexes were stained for rabbit-collagen type II (Red). The cells were previously labeled with CFDA cell tracer (Green), and all samples were stained with DAPI to identify the DNA strands of the nuclei. CAP, cell aggregate-fibrin-PLCL scaffold complex; SCP, single cell-fibrin-PLCL scaffold complex.

BMSCs within the whole cells-scaffold constructs were labeled with CFDA cell tracer (Green) and cultured for 7 days or 21 days in vitro, and then the cellular organization and differentiation were examined. Immunofluorescence staining with rabbit-collagen type II (Red) and DAPI (blue) shows a clear distinction between the CAPs and SCPs. Chondrogenically differentiated BMSCs in the lacunae that are surrounded by labeled type II collagen are seen in Figure 6. The differentiation of BMSCs in the cell aggregate was already ongoing on Day 7; thus, red fluorescence for collagen type II secreted by the differentiated chondrocytes was observed (Figure 6C), whereas the fluorescence was hardly observed in the SCPs (Figure 6A). Over time, as the BMSCs differentiated, the deposition of the labeled collagen type II increased and was more clearly observed in the newly formed tissues of the CAPs (Figure 6D) than in the SCPs (Figure 6B) on Day 21. Although DAPI staining showed homogeneously distributed nuclei of the cells in the SCPs and CAPs, some of the cell aggregates in the CAPs maintained their spherical morphologies after 21 days (Figure 6D).

**Figure 7:**
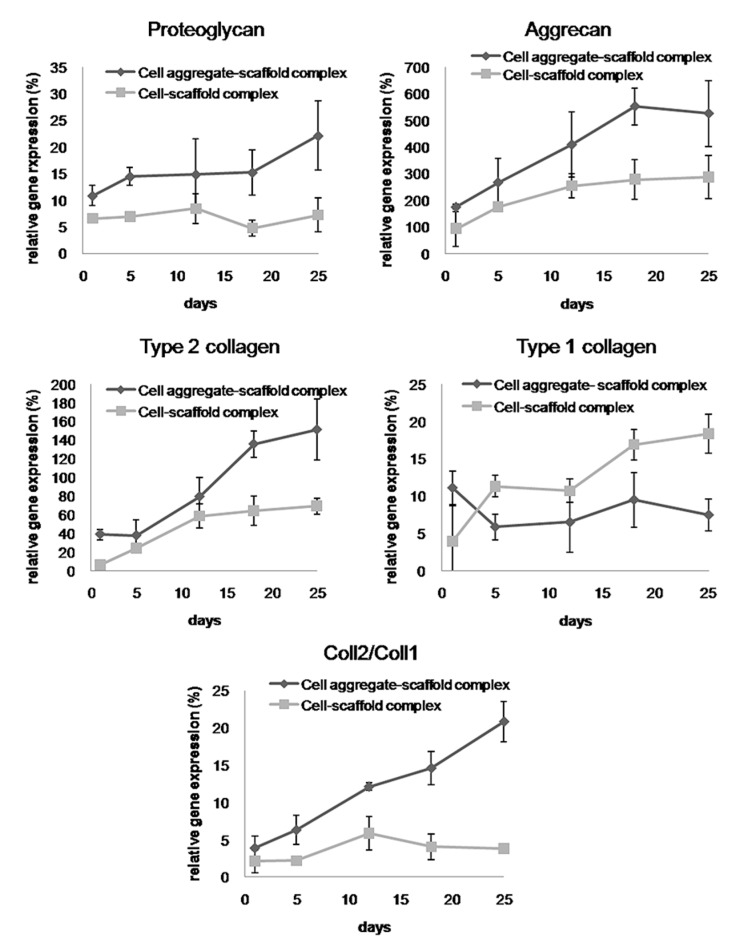
Relative mRNA expression of proteoglycan, aggrecan, type II collagen, type I collagen, and type II collagen to type I collagen (Coll2/Coll1) of the cell-fibrin-PLCL scaffold complexes and cell aggregate-fibrin-PLCL scaffold complexes cultured in vitro for up to 25 days. Error bars denote the standard deviation.

To investigate the differentiation of the seeded BMSCs, the expression of mRNA for proteoglycan, aggrecan, type II collagen, and type I collagen was assessed by real-time PCR (Figure 7). It revealed that the expression of various mRNAs tended to increase over time in both of the cells-scaffold complex groups; however, the CAPs had a much higher expression of proteoglycan, aggrecan, and type II collagen mRNAs that that of the other group. Moreover, the ratio of type II collagen to type I collagen expression, which is an important indicator of chondrogenic differentiation, was found to be significantly greater in the complexes that were seeded with cell aggregates than in those seeded with single cells. These results showed that seeding the cell aggregates into the fibrin-PLCL scaffold promoted the upregulation of chondrogenesis marker genes and chondrogenic differentiation in vitro.

**Figure 8:**
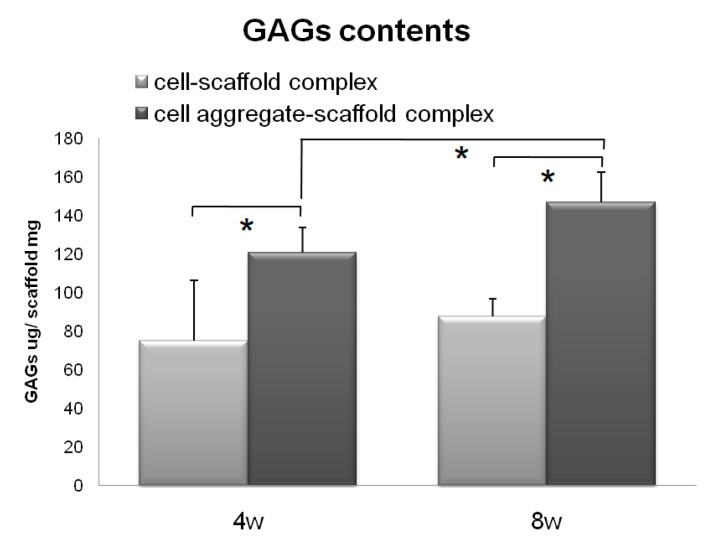
GAG levels within the cells-scaffold complexes explanted from nude mice at 4 and 8 weeks. (* = *p* < 0.05). Error bars denote the standard deviation.

Subsequently, the CAPs and SCPs were implanted subcutaneously into nude mice for 4 or 8 weeks in vivo for preliminary observation to determine which construct has a greater capacity for chondrogenic differentiation and cartilaginous tissue formation [42]. The results of the assay for 4 and 8 weeks are presented in Figure 8. Both the SCPs and CAPs showed increased GAG contents over time; however, the GAG contents of the CAPs increased from 120.90 ± 12.84 µg/mg scaffold at 4 weeks to 146.86 ± 31.12 µg/mg scaffold at 8 weeks, while those of the SCPs only increased from 75.30 ± 15.84 µg/mg scaffold at 4 weeks to 87.80 ± 9.20 µg/mg scaffold at 8 weeks. These results show that the GAG contents of the CAPs were much higher than those of the other complexes that were seeded with single cells. These trends were consistent with those obtained from the histological studies in terms of the accumulation of the cartilaginous extracellular matrix.

**Figure 9:**
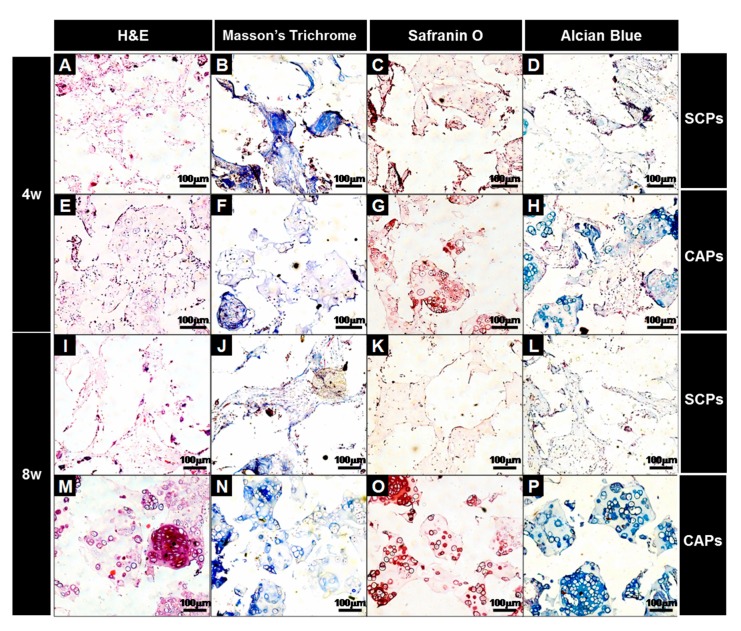
Histological studies of the implants at 4 (**A**–**H**) and 8 (**I**–**P**) weeks. The sections were stained with H&E (**A**,**E**,**I**,**M**), Masson’s Trichrome (**B**,**F**,**J**,**N**), Safranin O (**C**,**G**,**K**,**O**), or Alcian Blue (**D**,**H**,**L**,**P**). The images show the cell-fibrin-PLCL scaffold complexes (**A**–**D**,**I**–**L**) and the cell aggregate-fibrin-PLCL scaffold complexes (**E**–**H**,**M**–**P**).

A histological evaluation of the implants was carried out at the end of 4 and 8 weeks for the in vivo culture, and the images of each specimen stained with H&E, M-T, Safranin-O and Alcian blue are shown in Figure 9. H&E staining showed that cell aggregates in the CAPs formed mature and well-developed cartilaginous tissues close to native cartilage, evident by the chondrocytes within the lacunae. With Masson’s trichrome, Safranin-O, and Alcian blue stain, a substantial amount of collagen and homogeneously distributed sulfated GAGs, which were present in the extracellular matrices produced by the differentiated BMSCs in the newly-formed tissues, were observed in the CAPs. Additionally, although no significant difference in the deposition of collagen was present between those two groups at 4 weeks, lacunae were partially observed in the CAPs. Additionally, the presence of more lacunae and more accumulation of sGAGs were observed in the CAPs, with strong positive staining for M-T, Safranin-O, and Alcian blue at 8 weeks. The staining and lacunae were distributed homogeneously throughout the entire tissues, and the cells within the tissues also exhibited a cartilage-like morphology. In contrast, in the case of the SCPs, there was no significant change in the sGAG contents over time, and lacunae were never or rarely detected at 4 and 8 weeks. The results of the tissue histology show that the cell aggregate promoted the BMSCs to differentiate into chondrocytes, secrete the cartilaginous extracellular matrix, and form mature cartilage tissue.

**Figure 10:**
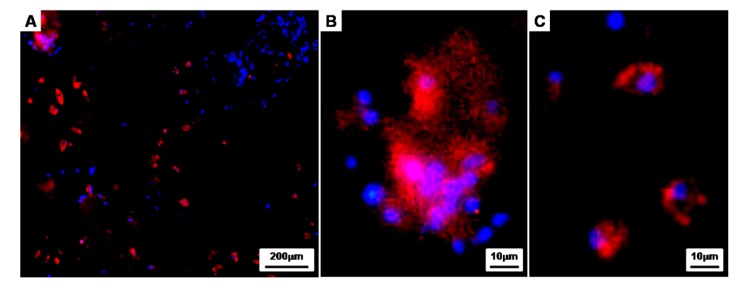
Immunofluorescence studies of the implants of cell aggregate-fibrin-PLCL scaffold complexes at 8 weeks. The sections were stained for rabbit-collagen type II (Red) and stained with DAPI to identify the DNA strands of the nuclei. Then, the images were merged to examine the chondrogenically differentiated BMSCs in the lacunae surrounded by the labeled type II collagen.

To further investigate the properties of the CAPs cultured for 8 weeks in vivo, immunofluorescence staining was conducted with Rabbit collagen type II (Red) and DAPI (blue) as a counterstain (Figure 10). Immunohistochemistry showed that the chondrogenically differentiated BMSCs within the lacunae were surrounded by labeled type II collagen (Red), and the collagen type II, an extracellular matrix material produced by differentiated chondrocytes, was clearly observed in the newly-formed tissues of the CAPs. Moreover, the magnified images show that the cell aggregates seeded onto the fibrin-PLCL scaffold were maintaining an aggregate shape (Figure 10A,B), and even single cells, which had migrated from the cell aggregate during differentiation, were also maintaining the chondrogenic phenotype (Figure 10C).

**Figure 11:**
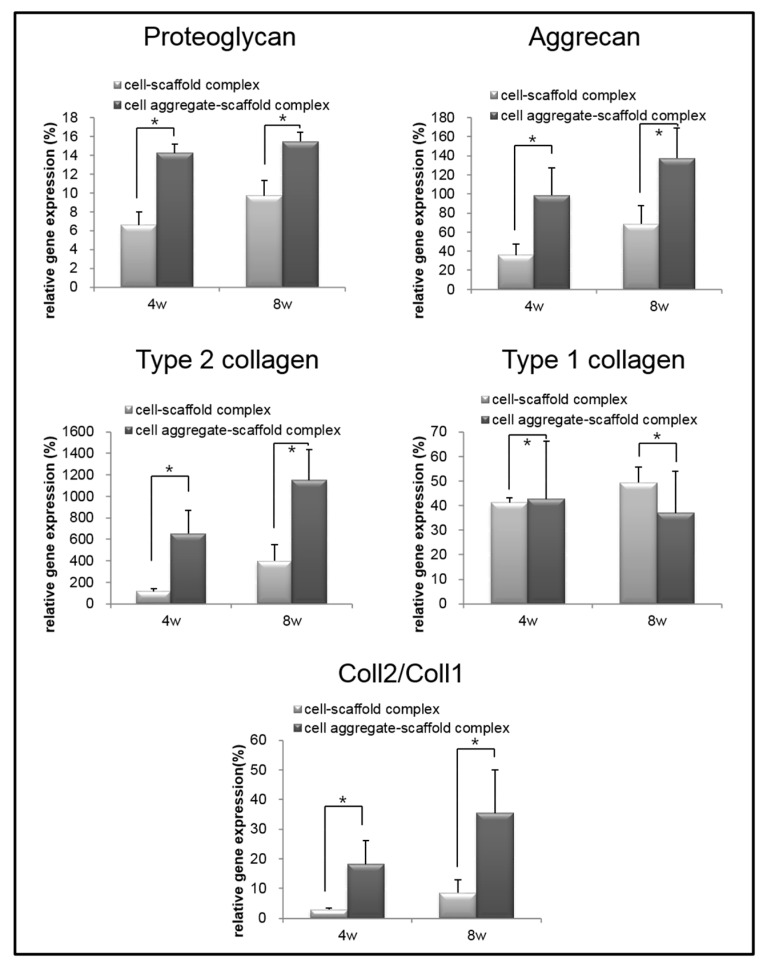
Relative mRNA expression of proteoglycan, aggrecan, type II collagen, type I collagen, and type II collagen to type I collagen for the cell-fibrin-PLCL scaffold complexes and cell aggregate-fibrin-PLCL scaffold complexes explanted at 4 and 8 weeks (* = *p* <0.05). Error bars denote the standard deviation.

To analyze the mRNA expression of proteoglycan, aggrecan, type II collagen, and type I collagen in the specimens retrieved after 4 and 8 weeks, real-time PCR was performed (Figure 11). The mRNA expression of proteoglycan, aggrecan, and type II collagen within the cells-scaffold complexes increased over time. However, a significant expression of cartilage-specific markers for the CAPs was observed over time compared to that of the SCPs.

Conversely, the expression of type I collagen gene in the CAPs decreased over time. In contrast, the expression of the type I collagen gene was increased in the SCPs. Besides, the ratio of type II collagen to type I collagen expression, which is an important indicator of chondrogenic differentiation, was found to be significantly greater in the complexes that were seeded with cell aggregates than in those seeded with single cells. These results show that cartilage-specific genes are expressed much more highly in the CAPs when compared to the SCPs. In other words, seeding cell aggregates onto the fibrin-PLCL scaffold facilitated the upregulation of chondrogenesis marker genes, the secretion of chondral extracellular matrix, and chondrogenic differentiation in vivo. The results of the in vivo experiments confirmed that the cell aggregates promoted themselves to secrete cartilaginous extracellular matrix, and to form mature cartilage tissue compared with that of the single cells. Moreover, the results show the effect of cell aggregates, which affect phenotype maintenance, tissue development, and chondrogenic differentiation.

## 4. Conclusions

In cartilage regeneration using stem cells, it is important to induce and maintain the differentiation of stem cells to cartilage lineages. Cell aggregates or speroids which are pre-directioned for chondrogenic differentiation enhance cartilaginous tissue formation. In this study, we prepared cell aggregates using the hanging drop method and evaluated the effect of the cell aggregates in hybrid scaffolds of fibrin gels and PLCL scaffolds for the chondrogenic differentiation of BMSCs, the production of GAGs, and the formation of cartilaginous tissue. From the in vitro and in vivo studies, significantly increased amounts of GAG contents and chondral extracellular matrix, and increased gene expression, were observed in the CAP, in which mature and well-developed cartilaginous tissues were formed. Overall, these results show that cell aggregates in hybrid scaffolds can promote themselves to differentiate into chondrocytes, can maintain their phenotypes, can enhance GAG production, and can form quality cartilaginous tissue, thereby inducing the effective regeneration of cartilage tissue close to natural cartilage in vitro and in vivo.

## Figures and Tables

**Table 1:** List of primers used in the real time polymerase chain reaction (PCR) analysis of the in vitro and in vivo samples.

Primer Name	Forward Sequence	Reverse Sequence	Product Size (bp)
Aggrecan	TCGAGGACAGCGAGGCC	TCGAGGGTGTAGGCGTGTAGAGA	94
Proteogly-can	CACCTACCAGGACAAGGT	GCGCAGGCTCTGGATCTC	78
Type II collagen	CCTGTGCGACGACATAATCTGT	GCAGTGGCGAGGTCAGTAG	98
Type I collagen	GGGTTTAGACCGTCGTGAGA	TTGCCAGGAGAACCAGCAAGA	170
GAPDH	GCACCGTCAAGGCTGAGAAC	ATGGTGGTGAAGACGCCAGT	142
